# Optical Amplification at 1.5 µm in Er^III^ Coordination Polymer‐Doped Waveguides Based on Intramolecular Energy Transfer

**DOI:** 10.1002/advs.202401131

**Published:** 2024-06-19

**Authors:** Xiaowu Shi, Yi Man, Yan He, Hui Xu, Baoping Zhang, Daquan Yu, Zhuliang Lin, Ziyue Lv, Zhiyuan Zhao, Linqi Zhang, Yongjian Chen, Dan Zhang

**Affiliations:** ^1^ Fujian Key Laboratory of Ultrafast Laser Technology and Applications School of Electronic Science and Engineering (National Model Microelectronics College) Xiamen University Xiamen 361005 China; ^2^ Key Laboratory of Functional Inorganic Material Chemistry Ministry of Education School of Chemistry and Material Science Heilongjiang University Harbin 150080 China

**Keywords:** 1.5 µm, coordination polymer [Er(DBTTA)_3_(FDPO)]_n_, intramolecular energy transfer, optical amplification, polymer waveguides

## Abstract

9,9‐bis (diphenylphosphorylphenyl) fluorene (FDPO) and dibenzotetrathienoacene (DBTTA), are synthesized as the neutral and anionic ligands, respectively, to prepare the Er^III^ coordination polymer [Er(DBTTA)_3_(FDPO)]_n_. Based on the intramolecular energy transfer, optical gains at 1.5 µm are demonstrated in [Er(DBTTA)_3_(FDPO)]_n_‐doped polymer waveguides under excitations of low‐power light‐emitting diodes (LEDs) instead of laser pumping. A ligand‐sensitization scheme between organic ligands and Er^3+^ ions under an excitation of an ultraviolet (UV) LED is established. Relative gains of 10.5 and 8.5 dB cm^−1^ are achieved at 1.53 and 1.55 µm, respectively, on a 1‐cm‐long SU‐8 channel waveguide with a cross‐section of 2 × 3 µm^2^ and a 1.5‐µm‐thick [Er(DBTTA)_3_(FDPO)]_n_‐doped polymethylmethacrylate (PMMA) as upper cladding. The Er^III^ coordination polymer [Er(DBTTA)_3_(FDPO)]_n_ can be conveniently integrated with various low‐loss inorganic waveguides to compensate for optical losses in the C‐band window. Moreover, by relying on the intramolecular energy transfer and UV LED top‐pumping technology, it is easy to achieve coupling packaging of erbium‐doped waveguide amplifiers (EDWAs) with pump sources in planar photonic integrated chips, effectively reducing the commercial costs.

## Graphical abstract

Based on the intramolecular energy transfer, relative gains of 10.5 and 8.5 dB cm^−1^ at 1535 and 1550 nm respectively are demonstrated in Er^III^ coordination polymer [Er(DBTTA)_3_(FDPO)]_n_‐doped waveguides under excitations of light‐emitting diode instead of laser pumping. This study is expected to achieve the application of polymer waveguide amplifiers in planar photonic integration and facilitate low‐cost packaging of the waveguide and pump sources.

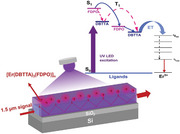

## 1 Introduction

Erbium‐doped optical waveguide amplifiers (EDWAs) can compensate for optical losses and play a key role in planar photonic integration. They are integrated with other active and passive optical waveguide devices, including arrayed waveguide gratings, micro‐ring resonators, and Mach–Zehnder modulators, to achieve an optical amplification of 1.5 µm. Conventionally, Er^3+^ ions are doped into various hosts as gain media for fabricating waveguide amplifiers. Examples include inorganic materials such as Al_2_O_3_,^[^ ^1^, ^2^ ^]^ silicates,^[^ ^3^, ^4^ ^]^ phosphates,^[^ ^5^, ^6^ ^]^ lithium niobate on insulators,^[^ ^7^, ^8^ ^]^ TeO_2_–ZnO,^[^ ^9^ ^]^ and GeO_2_–PbO glasses.^[^ ^10^ ^]^ Further, Er^3+^ ions can be used for synthesizing Er^III^ complexes with organic ligands, such as Er(DBM)_3_phen,^[^ ^11^ ^]^ Er_1.2_Yb_0.8_(PBa)_6_(Phen)_2_,^[^ ^12^, ^13^ ^]^ Er(THMD)_3_,^[^ ^14^ ^]^ and Er(DBTTA)_3_(DBFDPO),^[^ ^15^ ^]^ or be encapsulated into low phonon energy nanoparticles with Yb^3+^ ions, such as LaF_3_: Er^3+^, Yb^3+^,^[^ ^16^ ^]^ and NaYF_4_: Er^3+^, Yb^3+^.^[^ ^17^, ^18^, ^19^ ^]^ Finally, they were doped in polymer matrices for preparing polymer EDWAs. Compared with inorganic EDWAs, polymer EDWAs have many advantages, including simple processing methods, low cost, and easy integration with other photonic devices on various substrates. In the past 30 years, influenced by the research ideas of traditional erbium‐doped optical fiber amplifiers (EDFAs) and inorganic EDWAs, the 976 nm semiconductor lasers were typically selected as pump sources to provide optical pumping energy for polymer EDWAs. Er^3+^, Yb^3+^ co‐doped nanoparticles have been occupying the mainstream position in the research of polymer EDWAs attributed to their low phonon energy and high photoexcitation efficiency at 976 nm, while the development on polymer EDWAs based on Er^III^ complexes was almost stagnated.

Since the rare‐earth complex was proposed by Slooff in 2002,^[^ ^20^ ^]^ organic complexes were utilized for solving the solubility of inorganic rare‐earth ions in polymer matrices. However, the design of complexes with organic ligands is based on the fact that organic ligands have continuous absorption bands and large absorption cross‐sections (10^−22^–10^−23^ m^2^) in the 200–450 nm band.^[^ ^21^, ^22^ ^]^ The ultraviolet (UV) energy absorbed by organic ligands can be effectively transferred to the excited‐state level of the central rare‐earth ions via a sensitization mechanism. However, to the best of our knowledge, the advantages of organic complexes are yet to be maximized. Rare‐earth complexes are used to only solve the problem of the low solubility of rare‐earth ions in the polymer matrix. According to traditional research ideas, achieving an ideal optical gain performance at 1.5 µm in Er^III^ complexes is difficult because of the low absorption efficiency of Er^3+^ ions at 976 nm, gain quenching, and thermal damage to the polymer waveguide caused by the high power density of pump lasers.^[^ ^23^ ^]^

Based on the rapid development of gallium nitride‐based UV light‐emitting diodes (LEDs) light sources and broad industrial application prospects of rare‐earth complexes in the field of flat‐panel displays, we believe that developing a new research method that can fully utilize the advantages of rare‐earth complexes with strong UV absorption and intramolecular energy transfer between ligands and central rare‐earth ions can awaken the application of rare‐earth complexes in planar photonic integration. Therefore, in this study, 9,9‐bis (diphenylphosphorylphenyl) fluorene (FDPO) and dibenzotetrathienoacene (DBTTA), were synthesized as neutral and anionic ligands, respectively, to prepare coordination polymer [Er(DBTTA)_3_(FDPO)]_n_ featuring 1D chain structure. A ligand sensitization scheme between organic ligands DBTTA, FDPO, and Er^3+^ ions was established. Large continuous absorption bands of the organic ligands DBTTA and FDPO in the wavelength range of 300−420 nm were utilized to realize intramolecular energy transfer between the ligands and Er^3+^ ions under the excitation of low‐power UV LEDs. Relative gains of 10.5 and 8.5 dB cm^−1^ were achieved at 1.53 and 1.55 µm, respectively, on a 1‐cm‐long waveguide. The internal gain of 7.1 ± 0.3 dB cm^−1^ at 1.53 µm was achieved after deducting propagation losses.

Relying on the intramolecular energy transfer and LED top‐pumping, this study did not consider the constraints of traditional research ideas on EDFAs and EDWAs. It is expected to achieve the application of polymer EDWAs in planar photonic integration and facilitate low‐cost packaging of EDWAs and pump sources.

## 2 Experimental Section

### 2.1 Synthesis of Er^III^ Coordination Polymer [Er(DBTTA)_3_(FDPO)]_n_

**Figure 1:**
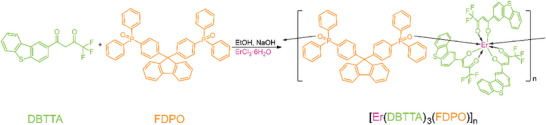
Molecular structure and synthetic procedure of coordination polymer [Er(DBTTA)_3_(FDPO)]_n_.

The coordination of Er^3+^ should keep stable during waveguide device fabrication, therefore ligands with strong coordination abilities, e.g. *β*‐diketone and phosphine oxide, were chosen as anionic and neutral ligands, respectively. More importantly, the aromatic ligands could not only improve absorption ability but also enhance molecular rigidity and steric hindrance. Therefore, excited‐state quenching by vibrational de‐excitation and inter‐polymer interactions could be alleviated. The coordination polymer [Er(DBTTA)_3_(FDPO)]_n_ was synthesized as per the process shown in **Figure** **1**. DBTTA (976 mg, 3 mmol) was dissolved in ethanol and NaOH (120 mg, 3 mmol) in a water solution (2 M) was added to remove the H^+^ in the DBTTA molecule. ErCl_3_·6H_2_O (382 mg, 1 mmol) in a water solution was added dropwise and the mixture was stirred at 60 °C for 30 min. Further, FDPO (719 mg, 1 mmol) in ethanol was added dropwise and the mixture was stirred at 60 °C for 4 h to afford the complex as a light‐pink precipitate. The purification was accomplished by precipitation from concentrated ethanol and water solutions, with a yield of 66%. Elemental analysis: Anal Calcd for C_97_H_60_F_9_ErO_8_P_2_S_3_: C 62.98, H 3.27, and S 5.20; found: C 62.62, H 3.32, and S 5.36. Fourier transform infrared spectroscopy (KBr pellet, cm^−1^): 3061, 1644, and 1625 (C═O stretching); 1547 and 1534 (C═C stretching in DBTTA); 1515, 1492, and 1437 (C─P stretching); 1322, 1289, 1220, 1186, and 1136 (P═O stretching).

Herein, an Er^3+^ coordination polymer was constructed featuring a 1D chain of Er(DBTTA)_3_ units bridged by bidentate phosphine oxide ligands. Compared to the reported Er^III^ complexes so far, which were almost mononuclear complexes used for polymer EDWAs fabrication, a coordination chain with appropriately concentrated Er^3+^ units not only ensured their uniform dispersion in the polymer matrix, but also increased the local Er^3+^ ions density, further enhancing the synergistic effects of ligands absorption, ligand‐to‐Er^3+^ energy transfer, and emissions from all Er^3+^ ions.

### 2.2 Preparation of Er^III^ Coordination Polymer‐Doped PMMA Films

0.01 g of [Er(DBTTA)_3_(FDPO)]_n_ powder was dissolved in 0.2 mL of tetrahydrofuran (THF), and was subsequently added to a solution of 1 g of polymethylmethacrylate (PMMA) in 4 mL of n‐butyl acetate. The concentration of [Er(DBTTA)_3_(FDPO)]_n_ was 1.0 wt.%. The resulting solution was stirred magnetically for 24 h to obtain a transparent solution. This solution was heated at 120 °C for 6 h to form a 200‐µm‐thick transparent film, which was used to measure the absorption, excitation, and photoluminescence (PL) properties. Further, a passive PMMA film and a PMMA film doped with ErCl_3_·6H_2_O were prepared for comparison. The passive PMMA film involved heating a solution of 1 g PMMA in 4 mL n‐butyl acetate at 120 °C for 6 h. For the PMMA film doped with ErCl_3_·6H_2_O, 0.01 g ErCl_3_·6H_2_O powder was dissolved in 0.2 mL ethanol and added to a solution of 1 g of PMMA in 4 mL n‐butyl acetate, which was then heated at 120 °C for 6 h. The thicknesses of the passive PMMA film and PMMA film doped with ErCl_3_·6H_2_O were 100 and 130 µm, respectively.

### 2.3 Absorption Properties

**Figure 2:**
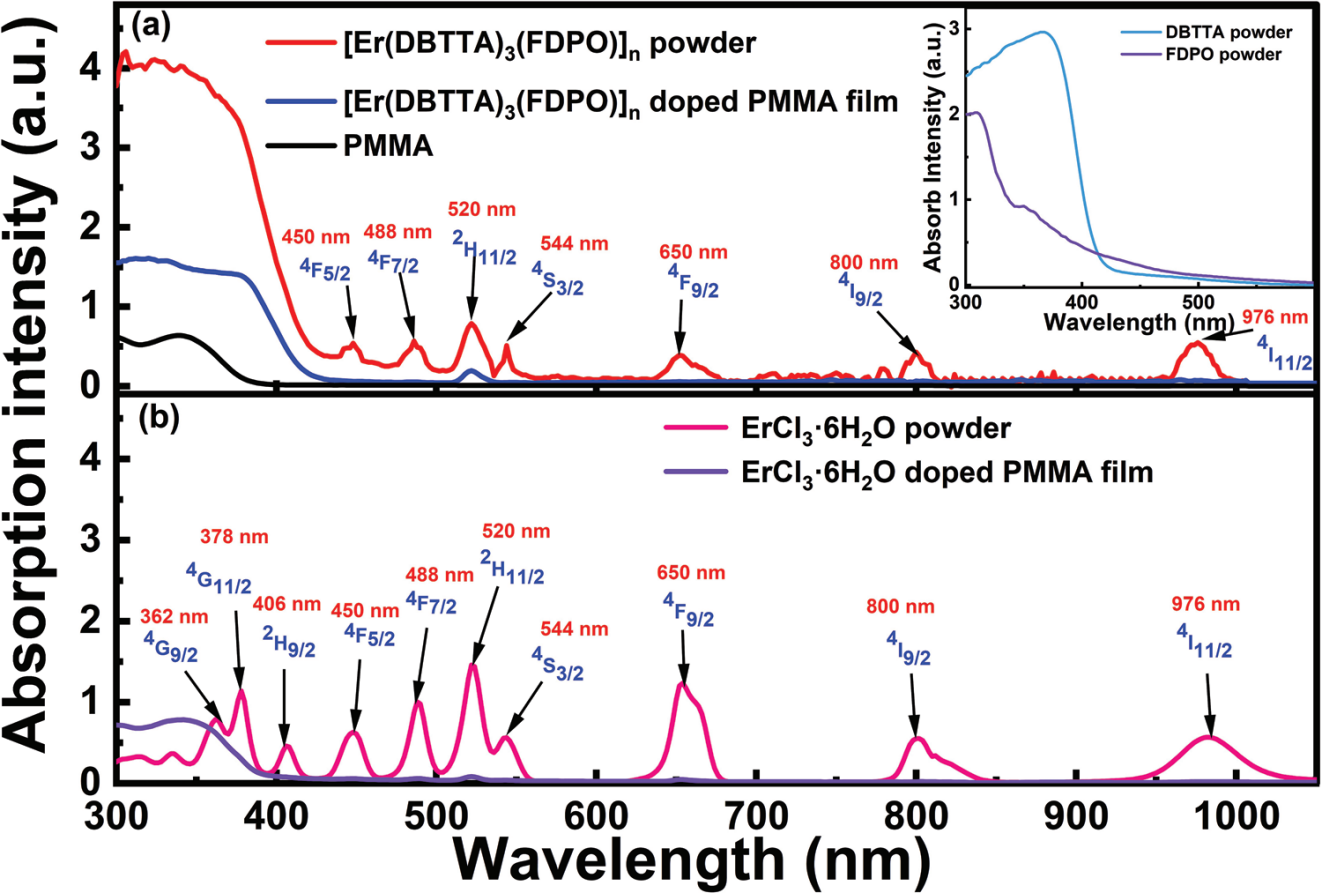
a) Absorption spectra of [Er(DBTTA)_3_(FDPO)]_n_ powder, [Er(DBTTA)_3_(FDPO)]_n_‐doped PMMA film, and PMMA film. The inset shows the absorption spectra of the organic ligands, DBTTA, and FDPO powder. b) Absorption spectra of ErCl_3_·6H_2_O powder and ErCl_3_·6H_2_O‐doped PMMA film.

**Figure** **2** shows the absorption spectra of the [Er(DBTTA)_3_(FDPO)]_n_ and ErCl_3_·6H_2_O measured by a UV–Vis–NIR spectrophotometer at 25 °C, respectively. Both ErCl_3_·6H_2_O and [Er(DBTTA)_3_(FDPO)]_n_ powders exhibited strong absorption peaks at 450, 488, 520, 544, 650, 800, and 976 nm, which corresponded to the intrinsic transitions of Er^3+^ ions from ground state ^4^I_15/2_ to the ^4^F_5/2_, ^4^F_7/2_, ^2^H_11/2_, ^4^S_3/2_, ^4^F_9/2_, ^4^I_9/2_, and ^4^I_11/2_ excited states, respectively. Compared to the UV absorption band of ErCl_3_·6H_2_O powder, [Er(DBTTA)_3_(FDPO)]_n_ exhibited a strong continuous absorption band in the 300–420 nm wavelength range because of the absorption of the ligands DBTTA and FDPO, as shown in the inset of Figure 2a. The absorption intensity of almost all intrinsic absorption peaks of Er^3+^ ions in the [Er(DBTTA)_3_(FDPO)]_n_‐doped PMMA film was significantly weaker than that of the [Er(DBTTA)_3_(FDPO)]_n_ powder because of the considerably lower doping concentration of Er^3+^ ions in the film. Only the intrinsic absorption of Er^3+^ ions at 520 nm corresponding to the ^4^I_15/2_ → ^2^H_11/2_ state could be indicated in the [Er(DBTTA)_3_(FDPO)]_n_‐doped PMMA film in Figure 2a. However, the doped film exhibited strong absorption in the UV range because of the absorption characteristics of the ligands DBTTA, FDPO, and PMMA within the 300–420 nm band, which implied that radiative transition luminescence of Er^3+^ ions could be achieved as long as there is an effective energy transfer effect between the organic ligands, PMMA host, and Er^3+^ ions if a UV band pump source is used for excitation.

### 2.4 Excitation and Photoluminescence Properties

**Figure 3:**
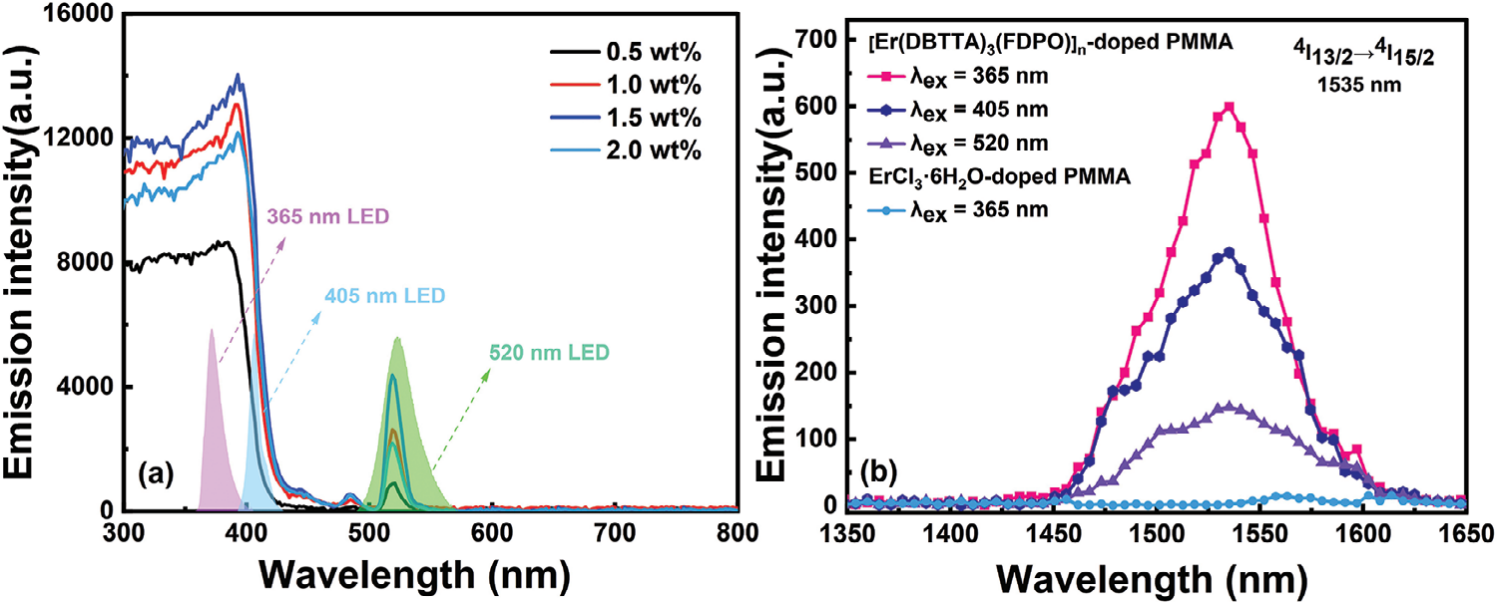
a) Excitation spectra of the [Er(DBTTA)_3_(FDPO)]_n_‐doped PMMA film with different concentrations at 25 °C; b) Near‐infrared PL spectra of ErCl_3_·6H_2_O‐ and [Er(DBTTA)_3_(FDPO)]_n_‐doped PMMA films under excitation of 365, 405, and 520 nm LEDs with the same power.

**Figure** **3** **a** shows the excitation spectra of the [Er(DBTTA)_3_(FDPO)]_n_‐doped PMMA films with different concentrations from 300–800 nm band, with an emission wavelength at 1535 nm (measured using an Edinburgh FLS1000 Steady‐state and transient fluorescence spectrometer). Light sources with wavelengths ranging from 300–420 nm and 520 nm can excite the [Er(DBTTA)_3_(FDPO)]_n_‐doped PMMA film to achieve the radiative transition luminescence of Er^3+^ ions at 1535 nm. According to the absorption spectra in Figure 2, the strong excitation effect of the UV band implied that the Er^III^ coordination polymer could establish an efficient internal energy‐transfer channel between the organic ligands and central Er^3+^ ion after absorbing the UV band energy. The excitation effect of 520 nm was achieved through the intrinsic absorption of Er^3+^ ions from the ^4^I_15/2_ to the ^2^H_11/2_ state, radiative transition occurs from the ^4^I_13/2_ to the ^4^I_15/2_ state. Comparing the excitation effects of the UV band and visible light at 520 nm revealed that the former was significantly more effective, which indicates that the near‐infrared luminescence effect achieved by driving the energy‐transfer channel established by the organic ligands surrounding the Er^3+^ ions was considerably better than that achieved solely by the intrinsic absorption of Er^3+^ ions. Further, in the concentration range of 0.5–1.5 wt.%, the luminescence intensity increased with an increasing concentration of [Er(DBTTA)_3_(FDPO)]_n_ in the PMMA host. When the concentration further increased to 2.0 wt.%, the luminescence intensity weakened because of concentration quenching. Although a 1.5 wt.% concentration exhibited stronger luminescence, the film formation smoothness was worse than that of the 1.0 wt.% concentration. Atomic force microscopy (AFM) images with doping concentrations of 1.0 and 1.5 wt.% can be found in Figure S1 (Supporting Information). Considering the transmission and scattering losses generated when the active material forms waveguides, which has a negative effect on achieving optical gain, a concentration of 1.0 wt.% was selected for subsequent waveguide preparation experiments.

Based on the excitation spectra, two UV LEDs with center wavelengths of 365 and 405 nm were selected as pump sources, in addition to a visible LED with a central wavelength of 520 nm, which corresponded to the intrinsic absorption of Er^3+^ ions (^4^I_15/2_→^2^H_11/2_) used for near‐infrared PL comparison. The radiation spectra of LEDs are indicated in Figure 3a. Figure 3b shows the PL spectra of the [Er(DBTTA)_3_(FDPO)]_n_‐doped PMMA film excited by 365, 405, and 520 nm LEDs, with each LED maintaining the same irradiation power of ≈100 mW. The emission peak at 1535 nm was attributed to the transition of Er^3+^ ions from metastable state ^4^I_13/2_ to ground state ^4^I_15/2_. The PL intensity excited by the 365 nm LED was stronger than that excited by the 405 nm LED under the same pump power excitation, whereas the excitation effect at the 520 nm LED was the weakest, consistent with the results of the excitation spectra shown in Figure 3a. The full width at half maximum (FWHM) remained almost unchanged at ≈70 nm. Meanwhile, almost no near‐infrared luminescence of Er^3+^ ions was observed with an excitation of the 365 nm LED in the ErCl_3_·6H_2_O‐doped PMMA film; however, there was an absorption band in the 300–400 nm range including the intrinsic absorption of Er^3+^ ions (^4^I_15/2_→ ^4^G_9/2_) and absorption of the PMMA host, as shown in Figure 2b. This indirectly proved that organic ligands could protect Er^3+^ ions from the influence of intermolecular interactions, and enhance molecular rigidity and steric hindrance, thereby reducing excited‐state quenching caused by vibration de‐excitation and inter‐polymer interactions. Here, PMMA could be considered as a doping matrix without energy transfer with Er^3+^ ions, and the intrinsic absorption (^4^I_15/2_ → ^4^G_9/2_) of Er^3+^ ions also failed to generate radiation transitions (^4^I_13/2_→^4^I_15/2_) in the film under the excitation of the UV LED. Thus, the emission of Er^3+^ ions in the [Er(DBTTA)_3_(FDPO)]_n_‐doped PMMA film could be almost entirely attributed to the organic ligands, DBTTA and FDPO, thereby providing energy for the Er^3+^ ions through the intramolecular energy transfer.

**Figure 4:**
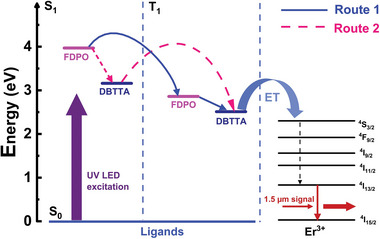
Schematic of energy‐transfer processes between the organic ligands DBTTA, FDPO, and Er^3+^ ions. S_1_: First‐excited singlet state; T_1_: First‐excited triplet state; ET: Energy transfer.

Based on the energy levels of the ligands DBTTA, FDPO, and Er^3+^ ions, as presented in **Table** **1**, a ligand sensitization scheme between the organic ligands and Er^3+^ ions is shown in **Figure** **4**. Both the singlet excited state S_1_ and triplet excited state T_1_ of the neutral ligand FDPO and anionic ligand DBTTA were higher than the lowest excitation state ^4^I_13/2_ of the Er^3+^ ion, which allows energy to transfer from the ligands to the central Er^3+^ ions. Under excitation by a UV LED, both FDPO and DBTTA could be excited from ground state S_0_ to the first excited state S_1_, and two energy‐transfer paths could be established to achieve synchronous energy‐transfer processes. First, the energy transferred from S_1_ of FDPO could be transferred to its own T_1_ state, and then, it was effectively transferred to T_1_ of DBTTA, as shown in Route 1. Energy could also be transferred from S_1_ of FDPO to S_1_ of DBTTA, followed by transfer to the T_1_ state of DBTTA, as shown in Route 2. Energy from a higher level tended to be transferred to a closer energy level in the sensitization mechanism. The energy gap between the two levels was moderate. An energy gap that is too large or too small reduces the efficiency of the energy transfer.^[^ ^25^ ^]^ An energy gap should maintain at least 0.15 eV (1200 cm^−1^), with an optimal value of ≈0.25 eV (2000 cm^−1^),^[^ ^26^ ^]^ thereby suppressing the reverse energy‐transfer process and eliminating the need for phonons to assist in energy transfer. Among the high‐energy excited states of Er^3+^ ions, the levels close in energy to T_1_ of DBTTA (2.51 eV, 20245 cm^−1^) were excited states ^4^F_9/2_ (1.92 eV, 15455 cm^−1^), ^4^S_3/2_ (2.30 eV, 18583 cm^−1^), and ^2^H_11/2_ (2.40 eV, 19337 cm^−1^), respectively. The energy gap between ^4^F_9/2_ and T_1_ of DBTTA was as high as 4790 cm^−1^, whereas the energy level gap between ^2^H_11/2_ and T_1_ was 908 cm^−1^. The optimal acceptor level of Er^3+^ ions is the ^4^S_3/2_ state, which had an energy gap of 1662 cm^−1^ with the energy level T_1_ of DBTTA. According to Malta's selection rule,^[^ ^27^, ^28^, ^29^, ^30^, ^31^ ^]^ due to the quantum number difference *|∆J|* of the total angular momentum of excited state ^4^S_3/2_ of Er^3+^ ions and emission transition state, i.e., ground state ^4^I_15/2_ is 6, the energy transfer mechanism belonged to the Förster resonance energy transfer (FRET) mechanism, characterized by multipole interactions. After obtaining the energy transfer from the T_1_ of DBTTA, the Er^3+^ ion could complete non‐radiative transition processes from the ^4^S_3/2_ to the ^4^I_13/2_ state and a radiative relaxation process from the ^4^I_13/2_ to the ^4^I_15/2_ state,^[^ ^32^ ^]^ ultimately realizing the optical amplification at 1.5 µm with an excitation of the 1.5 µm signal laser. In addition, the coordination polymer [Er(DBTTA)_3_(FDPO)]_n_ had a ligand‐bridged multinuclear configuration, giving rise to locally concentrated Er^3+^ ions and heterogeneous Er^3+^ distributions in PMMA, effectively reducing the distances between Er–Er and inter‐ligand to a few nanometers, which was short enough to support effective FRET between adjacent coordination repeat units. Therefore, active energy migration in polymeric systems could lead to effective synergy effects of the entire coordination repeating unit in the excited energy absorption of all ligands, ligand‐to‐Er^3+^ energy transfer, and 1.5 µm emissions from all Er^3+^ ions.

**Table 1:** Singlet and triplet energy levels of organic ligands.

Ligand	Singlet energy level [eV]	Triplet energy level [eV]
FDPO^[^ ^24^ ^]^	3.97	2.86
DBTTA^[^ ^24^ ^]^	3.16	2.51

The two energy‐transfer paths between organic ligands and Er^3+^ ions can be summarized as

(1)

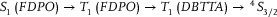



(2)

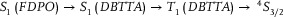




### 2.5 Waveguide Fabrication

**Figure 5:**
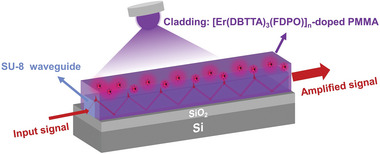
Propagation path of the evanescent wave and optical amplification process of the signal laser in the passive SU‐8 channel waveguide with the [Er(DBTTA)_3_(FDPO)]_n_‐doped PMMA as the upper cladding.

An evanescent‐field waveguide with the [Er(DBTTA)_3_(FDPO)]_n_‐doped PMMA polymer and a passive SU‐8 polymer as the active upper cladding and core, respectively, was designed and fabricated as shown in **Figure** **5**. The input optical signal laser could evanescently couple from the channel waveguide to the active upper cladding, accompanied by an LED placed above the waveguide to provide pump energy, which ultimately amplified the signal laser. Figure 5 shows the propagation path of the evanescent wave and optical amplification process on the surface of the channel waveguide. Compared with rectangular or embedded waveguide structures that use Er^3+^‐doped polymer materials as the core layer to form channel waveguides,^[^ ^14^, ^33^ ^]^ this waveguide structure allowed active polymers to spin‐coat different types of low‐loss waveguides easily, such as silicon on insulator (SOI), silicon nitride (Si_3_N_4_), fluorinated polymers, and glass, to compensate for optical losses by evanescent‐field coupling, significantly increasing their practicality in planar photonic integration.

**Figure 6:**
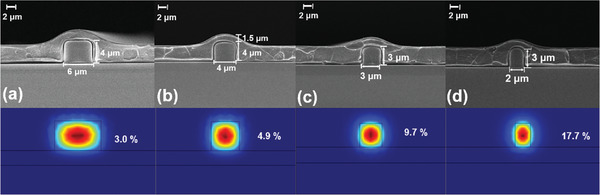
a–d) Cross‐sectional SEM micrographs and optical field distribution of TE mode profile at 1535 nm of evanescent‐field waveguides with four different cross‐sectional dimensions of 6 × 4, 4 × 4, 3 × 3, and 2 × 3 µm^2^, respectively. The SU‐8 polymer was used as the core and formed a channel waveguide via one‐step photolithography on a silicon substrate with 2‐µm‐thick SiO_2_. A ≈1.5‐µm‐thick [Er(DBTTA)_3_(FDPO)]_n_‐doped PMMA polymer was spin‐coated as the upper cladding.

The energy of the evanescent field obtained in the upper cladding was closely related to the cross‐sectional dimensions of the waveguide. Thus, four passive SU‐8 polymer waveguides with different cross‐sectional dimensions of 2 × 3, 3 × 3, 4 × 4, and 6 × 4 µm^2^ were fabricated by simple one‐step photolithography technology on a silicon substrate with a 2‐µm‐thick SiO_2_ layer. Then a ≈1.5‐µm‐thick [Er(DBTTA)_3_(FDPO)]_n_‐doped PMMA polymer was spin‐coated as the upper cladding. **Figure** **6** shows the scanning electron microscopy (SEM) micrographs of the waveguides with the four cross‐sectional dimensions. The optical field distribution of the signal laser at 1535 nm of TE mode profile in the evanescent‐field waveguide was simulated and analyzed using COMSOL Multiphysics software,^[^ ^34^ ^]^ as shown in Figures 6a–d. Proportions of evanescent‐field energy in the upper cladding of the waveguides with cross‐sectional dimensions of 2 × 3, 3 × 3, 4 × 4, and 6 × 4 µm^2^ were calculated to be ≈17.7%, 9.7%, 4.9%, and 3.0%, respectively. The smaller the cross‐section of the waveguide, the higher the energy of the evanescent field in the cladding, thereby resulting in a higher overlap integration factor between the signal laser and LED pump energy, which indicated better gain performance. The refractive indices of the SU‐8 and [Er(DBTTA)_3_(FDPO)]_n_‐doped PMMA polymer were measured to be 1.554 and 1.478 at 1535 nm using an ellipsometer (J. A. Woollam., Co. M2000), respectively.

## 3 Results and Discussion

**Figure 7:**
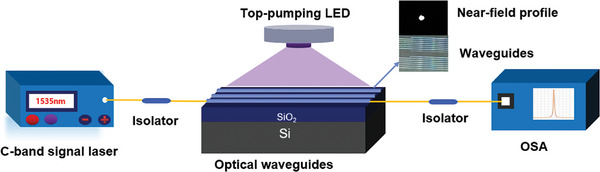
Experimental setup for measuring the optical gain of the polymer waveguide amplifier using the top‐pumping LED mode.

The experimental setup for the optical gain measurements was established using the vertical top‐pumping mode of the LED, as shown in **Figure** **7**. Lasers in the C bands of 1535 and 1550 nm were used as signal sources (KG‐DFB‐15‐10‐SM‐FA, Yuwei, China). The 365, 405, and 520 nm LEDs were selected as the pump sources. The LEDs are placed ≈2 mm above the waveguide. These LEDs have a viewing angle of 140° and a total illumination area of ≈1 cm^2^, and can almost completely cover the area of the 1‐cm‐long waveguide. When the LED power was 201 mW, the power available to the waveguide device was ≈181 mW. Thus, the radiation efficiency of the LED power collected by the device was ≈90% of the total LED power. The input‐signal laser was coupled to the waveguide using a single‐mode fiber with an isolator. The output signal was coupled with an optical spectrometer (Ocean Optics FLAME‐NIR‐INTSMA25). Unlike the conventional spatial beam coupling methods used for pumping EDWAs, which restrict the position of the waveguide to the 1D axial space,^[^ ^35^, ^36^, ^37^ ^]^ LED top‐pumping technology allows waveguides to be placed at any position in planar lightwave circuit, providing greater flexibility in photonic integration design and manufacturing. In addition, unlike the wavelength division multiplexer (WDM) coupling technology for coupling the 976 or 1480 nm pump laser and signal laser,^[^ ^16^, ^38^ ^]^ the up‐conversion effect of Er^3+^ ions and the destructive thermal damage to the waveguide caused by the high‐power laser to the waveguide end face can be effectively avoided. Moreover, the additional system loss introduced by WDM can be eliminated. A single LED can provide pump energy for an entire integrated optical circuit, thereby significantly reducing the commercial costs of EDWAs.

**Figure 8:**
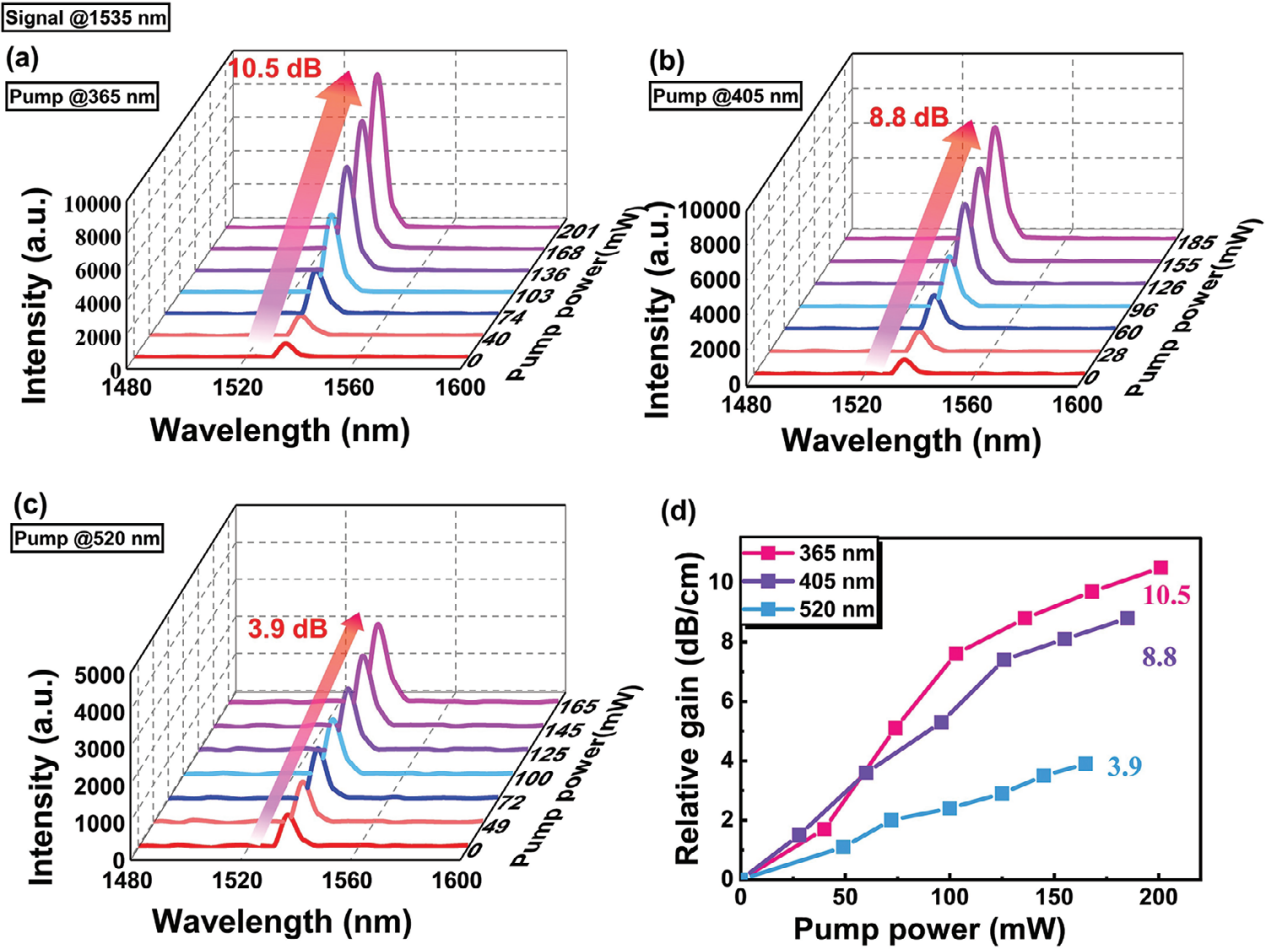
a–c) Output signal intensity at 1535 nm as a function of the pump power of the [Er(DBTTA)_3_(FDPO)]_n_‐doped PMMA based on an evanescent‐field waveguide under an excitation of 365, 405, and 520 nm LEDs, respectively. d) Comparison of relative gains as a function of pump power under three LEDs pumping conditions.

The relative gain is defined as $G_{rel}=10\mathrm{lg}\left(P_{S}^{Pump-on}/P_{S}^{Pump-off}\right)/L$, where $P_{S}^{Pump-on}$ and $P_{S}^{Pump-off}$ are the output signal powers measured by the optical spectrometer with and without pump power, respectively. **Figure** **8**a–c presents the relationship between the output optical signal intensity and the pump power under excitation of 365, 405, and 520 nm LEDs respectively in channel waveguides with a cross‐sectional dimension of 2 × 3 µm^2^. The waveguide length was 1 cm. The incident power of the signal laser at the waveguide input end face keeps 2.1 µW at 1535 nm. The maximum relative gains of the waveguide could reach 10.5, 8.8, and 3.9 dB cm^−1^ under excitation of 365 nm (201 mW), 405 nm (185 mW), and 520 nm LEDs (165 mW), respectively. Figure 8d compares the relationship between the relative gain and pump power under three LED excitations. The gains exhibit a linear growth trend with increasing pump power. At the same pump power, the relative gains obtained using 365 and 405 nm LED pumping were better than those obtained under 520 nm LED pumping, which is consistent with the conclusions from the excitation and PL spectra. In addition, owing to the weak absorption of the coordination polymer [Er(DBTTA)_3_(FDPO)]_n_ at 976 nm, it is difficult to achieve optical amplification at 1535 nm when a 976 nm semiconductor laser (200 mW) and the signal laser are coupled into the waveguide through a WDM.

**Figure 9:**
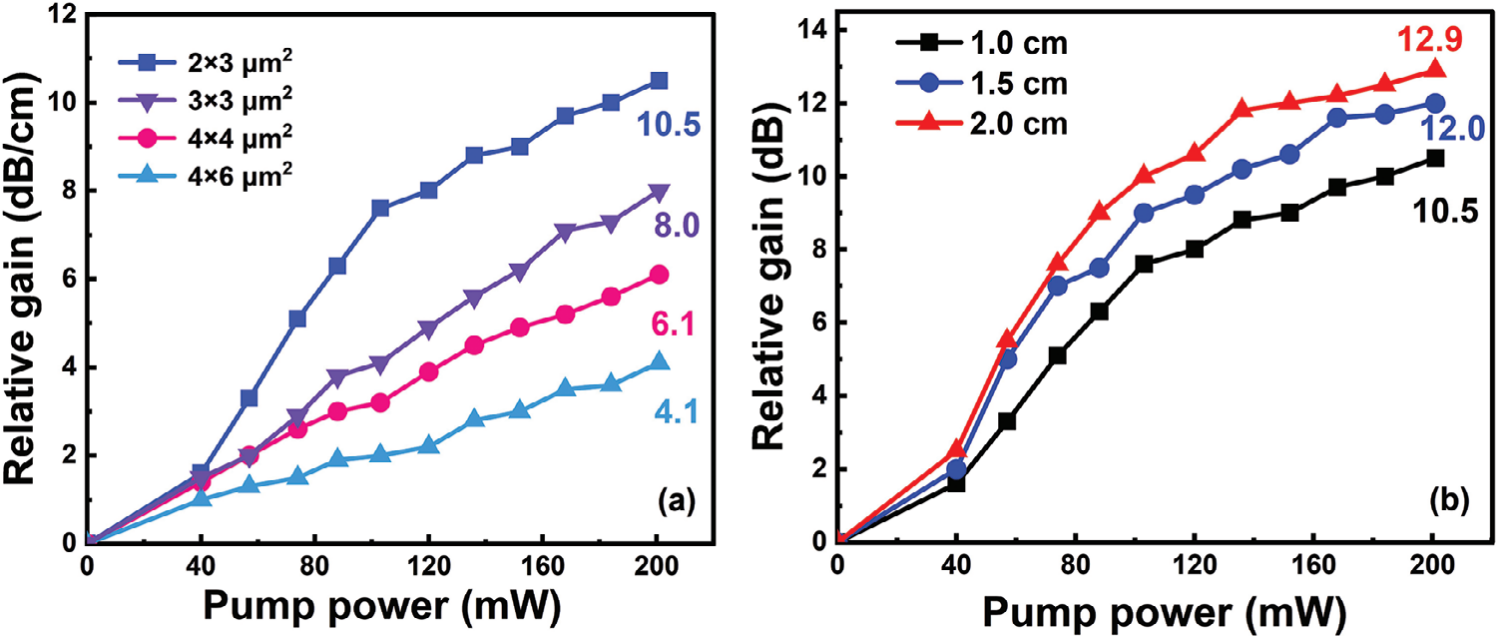
a) Comparison of relative gains in channel waveguides with different cross‐sectional dimensions. b) Relative gain of waveguides with different lengths.

The effect of the cross‐sectional dimensions of the channel waveguide on the gain is shown in **Figure** **9**a. The channel waveguides with cross‐sectional dimensions of 2 × 3, 3 × 3, 4 × 4, and 6 × 4 µm^2^ fabricated in Figure 6 were used for gain measurement. The waveguide length is maintained at 1 cm. When the input signal power is 2.1 µW, maximum gains of 10.5, 8.0, 6.1, and 4.1 dB were achieved in waveguides with cross‐sectional dimensions of 2 × 3, 3 × 3, 4 × 4, and 6 × 4 µm^2^ under the excitation of 201 mW 365 nm LED, respectively. The smaller the cross‐sectional dimension of the core, the higher the relative gain. According to the COMSOL Multiphysics software^[^ ^34^ ^]^ simulation results in Figure 6, for waveguides with cross‐sectional dimensions reduced from 6 × 4 to 2 × 3 µm^2^, the proportion of the signal laser escaping to the active upper cladding increased from 3.0% to 17.7%, and the gain value improved from 4.1 to 10.5 dB. Therefore, the evanescent wave and LED radiation light can achieve a higher overlap integration in the cladding when the signal laser is transmitted along the waveguide with a smaller core size, thereby achieving better gain performance. Further, the relative gain is related to the length of the waveguide. Figure 9b shows the relative gain of waveguides with different lengths. The gain increased linearly with the pump power for waveguides with lengths of 1.0, 1.5, and 2.0 cm, and the gain increased linearly with the pump power. Under the excitation of 201 mW 365 nm LED, gains of 10.5, 12.0, and 12.9 dB were observed for the above waveguides when the input signal light was 2.1 µW. The longer the waveguide (1–2 cm), the higher the relative gain (10.5–12.9 dB). This was attributed to an increase in the content of Er^3+^ ions in the long waveguide. However, the gain growth rate per centimeter does not vary linearly with the length of the waveguide. On the one hand, this is due to the influence of scattering and absorption losses of waveguides of different lengths. On the other hand, due to the fact that the radiation area of an LED pump source is concentrated on the device area of 1 cm^2^, as the waveguide becomes longer, the pump light cannot fully cover the waveguide, and the power required to sufficiently pump Er^3+^ ions also needs to be increased. Therefore, for the long waveguides, considering the use of LED array pumping is expected to further improve the gain.

**Figure 10:**
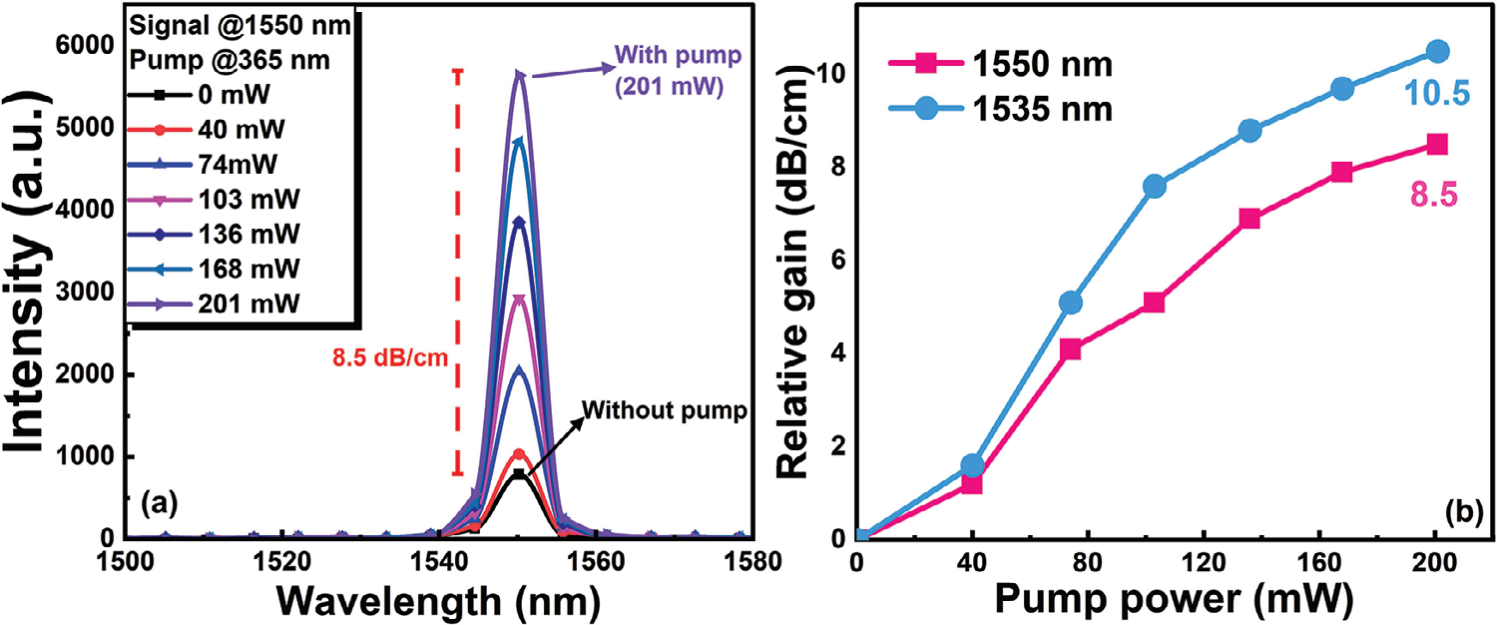
a) Output signal intensity at 1550 nm as a function of the pump power of the [Er(DBTTA)_3_(FDPO)]_n_‐doped PMMA waveguide under excitation of the 365 nm LED. b) Relationship between gain and pump power variations at wavelengths of 1535 and 1550 nm.

According to the PL spectra shown in Figure 3b, the FWHM of the [Er(DBTTA)_3_(FDPO)]_n_‐doped PMMA film was ≈70 nm. Different Er‐sites in gain media have an impact on the emission spectrum broadening of f–f transitions., e.g., Er^3+^ in phosphor–tellurite glass medium is broader than in bismuth silicate crystal.^[^ ^39^, ^40^, ^41^, ^42^ ^]^ Meanwhile, the coupling between the f–f electron transition and the C─H, O─H vibrational groups in the polymer matrix also contributes to the broadening of the emission spectrum.^[^ ^43^ ^]^ Such a wide FWHM indicated a wide gain band, thereby demonstrating the potential to provide gain in the third window of optical communication (1550 nm). The relationship between the output optical signal intensity at 1550 nm and the pump power under the excitation of the 365 nm LED was also demonstrated, as indicated in **Figure** **10**a. A relative gain of 8.5 dB was achieved in a 1‐cm‐long channel waveguide with a cross‐sectional dimension of 2 × 3 µm^2^ under an excitation of 201 mW 365 nm LED when the signal laser power at 1550 nm was 2.1 µW. Compared to the gains at 1535 nm, which is the peak emission wavelength of Er^3+^ ions, the relative gains at 1550 nm remained ≈2 dB lower as the pump power increased in the range of 100–200 mW, as shown in Figure 10b.

**Figure 11:**
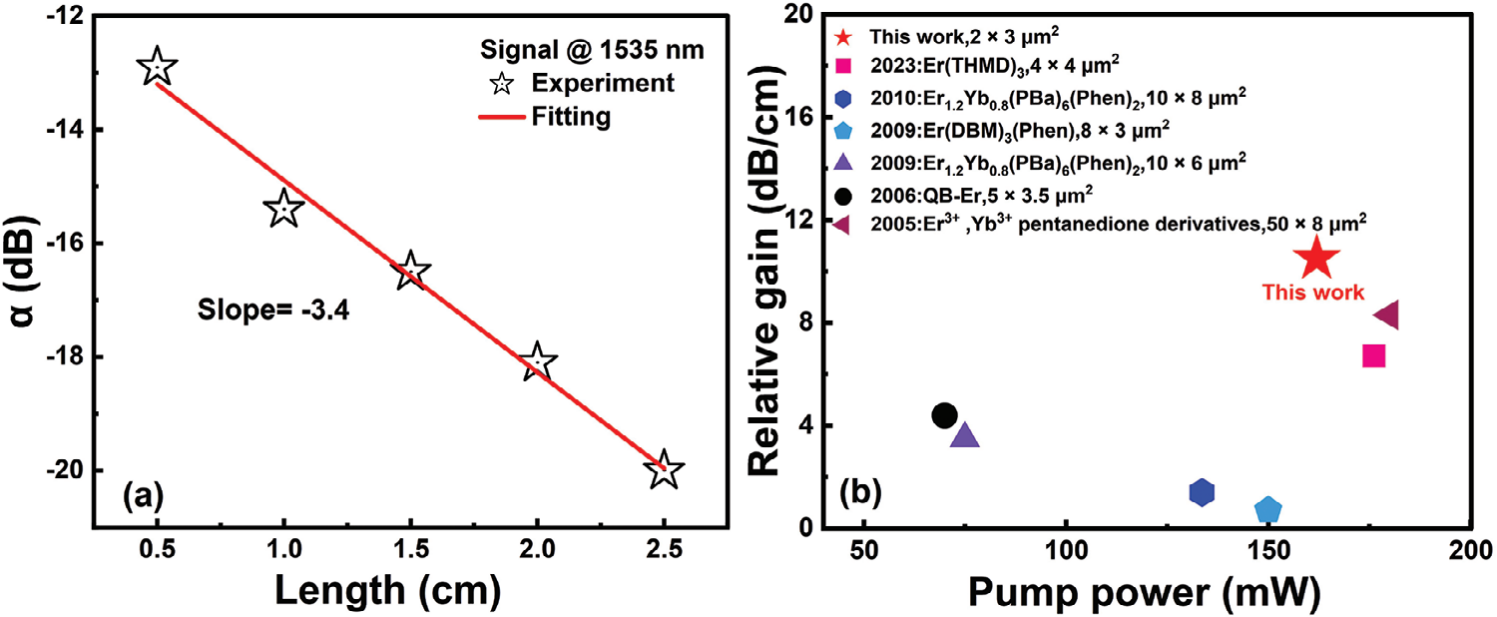
a) Propagation loss of the evanescent‐field waveguide with a cross‐section of 2 × 3 µm^2^ at 1535 nm. b) Summary of the relative gains of Er^III^ complex‐doped polymer waveguide amplifiers operating at the C band over recent years.

The relationship between the relative gain *G_rel_* and internal gain *G_int_* can be described by

(3) $$G_{int}=G_{rel}-\alpha_{loss}$$

where α_*loss*_ denotes the waveguide propagation loss. For the evanescent‐field waveguide with a cross‐section of 2 × 3 µm^2^, the propagation loss measured by the cutback method was ≈3.4 ± 0.3 dB cm^−1^ at 1535 nm, as illustrated in **Figure** **11**a. Thus, the value of *G_int_* was ≈7.1 ± 0.3 dB cm^−1^ at 1535 nm.

Over the past 30 years, the research route of fabricating polymer EDWAs by doping Er^III^ complexes has not been optimistic and it is about to be abandoned. The low absorption cross‐section and absorption efficiency at 976 nm, as well as the up‐conversion luminescence of Er^3+^ ions and the thermal damage of the polymer waveguide caused by high‐power laser pumping (100–400 mW), limit the development and application of the Er^III^ complex in the field of planar photonic integration. Wong et al.^[^ ^44^ ^]^ showed a relative gain of 8.3 dB cm^−1^ at 1533 nm in an Er^3+^, Yb^3+^ pentanedione derivatives‐co‐doped SU‐8 polymer waveguide with a cross‐sectional dimension of 50 × 8 µm^2^, which is the best‐known result. Thus far, the research methods for polymer EDWAs based on Er^III^ complex‐doped materials, including QB‐Er,^[^ ^45^ ^]^ Er(DBM)_3_Phen,^[^ ^11^ ^]^ and Er_1.2_Yb_0.8_(PBa)_6_(Phen)_2_^[^ ^12^, ^13^ ^]^ are similar, which almost follow the research approach of inorganic EDWAs, i.e., by utilizing the intrinsic energy levels of Er^3+^ ions pumped by traditional 976 or 1480 nm lasers. The gain level is maintained at <5 dB cm^−1^, as shown in Figure 11b. In this study, the advantages of the Er^III^ complex were fully utilized, and Er^3+^ coordination polymer featuring a 1D chain of Er(DBTTA)_3_ units bridged by bidentate phosphine oxide ligands with an efficient intramolecular energy transfer was designed. Combined with the low‐power UV LED top‐pumping technology, a relative gain and internal gain of ≈10.5 and 7.1 dB cm^−1^ was achieved in a 2 × 3 µm^2^ passive SU‐8 waveguide with ≈1.5‐µm‐thick [Er(DBTTA)_3_(FDPO)]_n_‐doped PMMA as the upper cladding. Moreover, the [Er(DBTTA)_3_(FDPO)]_n_‐doped polymer can be popularized on various types of substrates to compensate for the optical loss, including commercialized SOI waveguide (loss of 0.5 dB cm^−1[^ ^46^ ^]^), Si_3_N_4_ waveguide (0.25 dB cm^−1[^ ^47^ ^]^), and tellurite glass (0.9 dB cm^−1[^ ^48^ ^]^). By utilizing the amplification ability of the Er^III^ coordination polymer with a relative gain of 8.5 dB cm^−1^ at 1550 nm, and integrating with the three low‐loss waveguides mentioned above through evanescent wave coupling, it is expected to achieve internal gains of ≈8.0, 8.3, and 7.6 dB cm^−1^ respectively.

## 4 Conclusion

Phosphine oxide and β‐diketone ligands, FDPO and DBTTA, were prepared as neutral and anionic ligands respectively to synthesize Er^III^ coordination polymer [Er(DBTTA)_3_(FDPO)]_n_. The absorption, excitation, and PL properties of the active polymer were investigated. A ligand sensitization scheme involving two simultaneous routes between the organic ligands FDPO and DBTTA and Er^3+^ ions under UV LED excitation is discussed. Based on the intramolecular energy transfer, optical gains were demonstrated in polymer waveguides by utilizing three low‐power 365, 405, and 520 nm LEDs rather than the conventional 976 or 1480 nm semiconductor lasers as pumping sources. The relative gains of the 10.5 and 8.5 dB cm^−1^ were achieved at 1535 and 1550 nm, respectively, in a 1‐cm‐long SU‐8 waveguide with a cross‐section of 2 × 3 µm^2^ and ≈1.5‐µm‐thick [Er(DBTTA)_3_(FDPO)]_n_‐doped PMMA as the upper cladding. Using the cutback methods, the propagation loss of the waveguide at 1535 nm was measured to be ≈3.4 ± 0.3 dB cm^−1^, and the internal gain was determined to be ≈7.1 ± 0.3 dB cm^−1^. The effect of the cross‐sectional dimensions and length of the channel waveguide on the gain performance was also discussed. Relying on the intramolecular energy transfer and LED top‐pumping, this study did not consider the constraints of traditional research ideas on EDFAs and EDWAs, and it is believed to awaken the application of rare‐earth complexes in planar photonic integration.

## Conflict of Interest

The authors declare no conflict of interest.

## Supporting information

Supporting Information

## Data Availability

The data that support the findings of this study are available from the corresponding author upon reasonable request.
