# Supplementary material for: Optical Amplification at 1.5 µm in ErIII Coordination Polymer‐Doped Waveguides Based on Intramolecular Energy Transfer
Source: Adv Sci (Weinh). 2024 Jun 19;11(31):2401131. doi: 10.1002/advs.202401131 (PMC11336911; doi:10.1002/advs.202401131)
Supplement: Supplementary file 1 — Supporting Information [file ADVS-11-2401131-s001.docx]

Supporting Information

**Optical amplification at 1.5 µm in Er^Ⅲ^ Coordination Polymer-Doped Waveguides Based on Intramolecular Energy Transfer**

Xiaowu Shi, Yi Man, Yan He, Hui Xu,* Baoping Zhang, Daquan Yu, Zhuliang Lin, Ziyue Lv, Zhiyuan Zhao, Linqi Zhang, Yongjian Chen, and Dan Zhang*

Xiaowu Shi, Yan He, Baoping Zhang, Daquan Yu, Zhuliang Lin, Ziyue Lv, Zhiyuan Zhao, Linqi Zhang, Yongjian Chen, and Dan Zhang

Fujian Key Laboratory of Ultrafast Laser Technology and Applications, School of Electronic Science and Engineering (National Model Microelectronics College), Xiamen University, Xiamen, 361005, China

1. mail: zhangdan@xmu.edu.cn

Y. Man, H. Xu

Key Laboratory of Functional Inorganic Material Chemistry, Ministry of Education, School of Chemistry and Material Science, Heilongjiang University, Harbin, 150080, China

E-mail: hxu@hlju.edu.cn

X.S. and Y.M. contributed equally to this work.


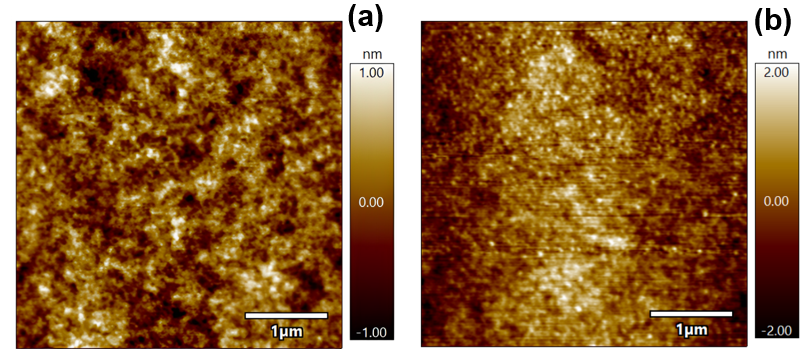


**Figure S1.** Atomic force microscopy (AFM) images of the [Er(DBTTA)_3_(FDPO)]_n_-doped PMMA films at different concentrations. (a) 1.0 wt.%; (b) 1.5 wt.%. Within a 4 × 4 μm^2^ area, the root mean square roughness *R*_q_ for the films with mass percentages of 1.0 and 1.5 wt.% were 0.34 and 0.65 nm, respectively. The corresponding arithmetic mean roughness *R*_a_ was 0.27 and 0.53 nm, respectively.
